# Abolition by design: implementing the dismantling of the commercial tobacco and nicotine industry

**DOI:** 10.1093/heapro/daag133

**Published:** 2026-10-14

**Authors:** Raglan Maddox, Shane Kawenata Bradbrook

**Affiliations:** Bagumani (Modewa) Clan, Papua New Guinea; Yardhura Walani, National Centre for Aboriginal and Torres Strait Islander Wellbeing Research, National Centre for Epidemiology and Population Health, Australian National University (ANU), College of Law, Governance and Policy, Canberra, ACT, Australia; Ngāi Tāmanuhiri, Rongowhakaata, Ngāti Kahungunu, Aotearoa, New Zealand

## Abstract

The commercial tobacco and nicotine industry continues to drive preventable disease and death globally. Despite progress in tobacco control, industry power, product diversification, market expansion, and policy interference have constrained the impact of incremental regulatory approaches. Downstream interventions, such as cessation supports and lung cancer screening, remain essential but respond to accumulated harms rather than preventing exposure at its source. This paper presents an implementation framework that positions abolition as a staged, governable, and legally supported public health strategy. Rather than a symbolic endpoint, abolition is conceptualized as a sequenced governance process through which industry structures can be dismantled using existing regulatory, fiscal, and enforcement mechanisms. These mechanisms are embedded within national systems and international instruments, including the World Health Organization Framework Convention on Tobacco Control and international human rights instruments. Abolition is mischaracterized as consumer prohibition. This reflects a category error that evaluates abolition using prohibition logics, obscuring its focus on dismantling corporate production, supply, and profit infrastructures through existing governance and regulatory tools. The framework conceptualizes the industry as an ecosystem spanning corporate structures, product categories, supply chains, science distortion, and political influence. Each domain is mapped to disruption levers and institutions, with the aim of reducing implementation risk and limiting industry adaptation. Abolition is framed as a coordinated governance project across local, national, and global levels, with Indigenous peoples and young people recognized as leaders shaping accountability, prevention pathways, and norms. This approach illustrates how abolition-oriented reform may be administratively feasible, legally defensible, and relevant to advancing health equity.

Contribution to Health Promotion

- Explains why reducing tobacco harms alone is insufficient, and why ending the commercial tobacco and nicotine industry should be considered a public health goal.
- Demonstrates how governments can use existing laws, taxation, and regulatory tools to progressively dismantle commercial tobacco and nicotine supply.
- Links tobacco control with health promotion principles by addressing the commercial determinants of health and strengthening the accountability of harmful industries.
- (Re)frames tobacco control from managing ongoing harm towards preventing future harm by addressing the commercial systems that sustain it.

## Introduction

The commercial tobacco and nicotine industry fuels preventable disease and premature death globally (Thurber *et al*. 2021, Joshy *et al*. 2025). Despite decades of tobacco control, substantial harm persists, particularly among Indigenous peoples and populations exposed to structural disadvantage and commercial targeting (Odo *et al*. 2025, Pousini-Hilton *et al*. 2025). Although comprehensive tobacco control has substantially reduced smoking prevalence in many jurisdictions, declining prevalence has not necessarily produced a corresponding reduction in corporate power or the capacity of commercial actors to generate new markets. Major tobacco companies have continued to reproduce commercial harm through product diversification, market expansion, corporate restructuring, and policy interference (Edwards *et al*. 2022, Gilmore *et al*. 2023, Maddox *et al*. 2026). These adaptive strategies enable commercial and political influence to continue even where conventional cigarette consumption is declining.

In Building Love and Justice, Ending Harms, we established abolition of the commercial tobacco and nicotine industry as an Indigenous-led, justice-based public health framework grounded in human rights, sovereignty, love and structural responsibility, and distinguished abolition from consumer prohibition (Maddox and Bradbrook 2025). The present paper addresses a different question: how abolition can be operationalized through existing governance, regulatory, and institutional systems. It therefore translates the earlier normative framework into a governance-oriented implementation framework identifying pathways through which abolition-oriented reform could proceed.

Despite increasing recognition of the commercial determinants of health, many tobacco-control frameworks continue to assume the long-term persistence of commercial nicotine markets. Abolition is consequently treated as aspirational or infeasible because it is often imagined as abrupt consumer prohibition rather than a staged governance process. This framing underestimates both the capacity of states and the fiscal, licensing, corporate, regulatory, and public health powers available to progressively constrain harmful commercial systems (Fagerstrom *et al*. 2025, Department of Parliamentary Services 2026).

Governments have previously phased out or severely restricted harmful products and related commercial activities through measures including licence withdrawal, production limits, market contraction, and product standards. Examples include asbestos, engineered stone, chlorofluorocarbons, leaded petrol, leaded paint, trans fats, and commercial sunbeds. These examples are not equivalent to full industry abolition, but they demonstrate that structural phase-out approaches can be implemented where harms are sufficiently severe and persistent (Proctor 2012, Maddox and Bradbrook 2025).

Tobacco endgame scholarship and more recent policy work have similarly expanded the range of measures considered possible, including supply reduction, retail contraction, and restrictions extending beyond the minimum requirements of the World Health Organization Framework Convention on Tobacco Control (WHO FCTC; Malone 2010, 2013, Thomson *et al*. 2012, McDaniel *et al*. 2016, Hefler 2025). Forward-looking measures under Article 2.1 have now also been considered through the formal Conference of the Parties (COP) process under the WHO FCTC, as discussed below.

At COP10, the COP adopted Decision FCTC/COP10(12), establishing an Expert Group to identify and describe forward-looking tobacco-control measures that could be contemplated within the scope of Article 2.1 of the WHO FCTC. The Expert Group subsequently reported to COP11 in document FCTC/COP/11/5, identifying 16 forward-looking measures. In Decision FCTC/COP11(5), COP11 welcomed the report and invited Parties, in accordance with their national laws, regulations, contexts, and priorities, to note the voluntary measures as options for consideration. The list is non-exhaustive, and the decision does not require Parties to implement the measures (Conference of the Parties 2024, 2025, Expert Group on Forward-looking Tobacco Control Measures 2025).

Endgame frameworks have expanded policy horizons but typically adopt prevalence thresholds (e.g. below 5%) that signal policy success while leaving residual industrial harm in place (Ait Ouakrim *et al*. 2024, Perusco *et al*. 2025a, 2025b). Abolition addresses this limitation by defining the commercial tobacco and nicotine industry as the explicit object of intervention and providing a more precise governance framework for structural transition towards eradicating industrialized tobacco and nicotine harms (Proctor 2012, Maddox and Bradbrook 2025).

In this paper, abolition refers to the phased dismantling of commercial structures that manufacture, market, and distribute tobacco and nicotine products at population scale. It does not require criminalization of individual use or assume the immediate elimination of demand (Proctor 2012, Maddox and Bradbrook 2025). Rather, abolition is conceptualized as a governance approach that progressively reduces commercial supply, limits industry influence, and realigns regulatory systems towards long-term eradication of industrialized tobacco and nicotine harms. Throughout this paper, ‘abolition’, ‘dismantling’, and ‘industry exit’ are used to describe the same staged governance process (Maddox and Bradbrook 2025).

Industry abolition describes the governance pathway, while the eradication of industrialized tobacco and nicotine harms represents the intended public health and well-being outcome. These harms include addiction, disease, premature death, commercial exploitation, policy interference, and the (re)production of inequities through commercial nicotine markets. Framed as upstream structural prevention, abolition aligns with health promotion’s longstanding commitment to addressing the commercial, political, and social determinants of health.

Throughout this paper, the term ‘commercial tobacco and nicotine industry’ is used as a broader analytical and implementation category encompassing commercial entities and associated infrastructures involved in the ownership, manufacture, financing, marketing, distribution, and retailing of tobacco products and non-therapeutic nicotine products, together with related political influence and knowledge-production systems. This usage is broader than the definition of ‘tobacco industry’ in Article 1 of the WHO FCTC, which refers to tobacco manufacturers, wholesale distributors, and importers of tobacco products (World Health Organization 2003). When discussing obligations arising specifically under the WHO FCTC, we therefore use the treaty term ‘tobacco industry’. The framework is directed towards commercial tobacco and non-therapeutic nicotine markets and does not seek to restrict approved cessation medicines supplied within therapeutic goods and medicine-regulation systems.

While emerging tobacco-control literature has called for policy adaptation and regulatory evolution, it largely assumes the persistence of commercial nicotine markets. The present paper engages this literature while shifting the analytical focus from market optimization to structural transition.

Major transnational tobacco companies have advanced narratives of corporate ‘transformation’, claiming that they are moving away from harmful products in response to declining cigarette consumption, regulatory pressure, and changing public expectations. Edwards *et al*. (2022) found little evidence that these claims represent substantive transformation away from business models dependent on tobacco and nicotine sales. Although genuine corporate transition remains theoretically possible, contemporary adaptation has predominantly involved product diversification, regulatory circumvention, market expansion, and the continuation of commercial nicotine dependence under new product and regulatory conditions (Legg *et al*. 2021, Edwards *et al*. 2022, Maddox *et al*. 2026). The framework therefore does not rely on voluntary transformation by transnational tobacco companies as a pathway to structural reform.

Drawing on the WHO FCTC and international human rights instruments, including the United Nations Declaration on the Rights of Indigenous Peoples, we present an operational framework for abolishing the commercial tobacco and nicotine industry as a staged, component-specific, and institutionally distributed process (World Health Organization 2003, United Nations 2007). The framework maps established regulatory and governance mechanisms to discrete components of the industry ecosystem, while recognizing that their application may require new or amended legislation. The availability, scope, and legal defensibility of specific levers will vary across jurisdictions according to constitutional arrangements, regulatory structures, political context, and state capacity.

We conceptualize abolition not as a single policy decision but as a process of deliberate system (re)design. This approach aligns with the incremental strengthening logic embedded within the WHO FCTC and associated decisions, while extending that logic towards structural resolution rather than perpetual mitigation (World Health Organization 2003, Maddox and Bradbrook 2025).

The paper proceeds by first clarifying why precise definition of the industry is a prerequisite for implementation. By understanding the commercial tobacco and nicotine industry as an interconnected and governable ecosystem, the paper specifies the object of intervention with sufficient precision to support enforcement, accountability, policy sequencing, and reform durability. It then outlines component-specific abolition and disruption levers and the institutions responsible for their enactment, before presenting a phased sequencing framework intended to guide, rather than prescribe, implementation. This sequencing framework aims to protect, insulate, and stabilize governance, regulate industry power, and enable durable exit over time. We conclude by addressing feasibility, legal defensibility, and governance considerations, demonstrating that abolition-oriented approaches may be compatible with existing governance functions and administrative systems across many jurisdictions.

## From framework to implementation: operationalizing abolition within existing governance systems

Building on the normative framework developed in Building Love and Justice, Ending Harms, this section specifies how abolition could be operationalized through existing regulatory, fiscal, enforcement, and accountability systems. The emphasis therefore shifts from establishing the ethical case for abolition to identifying the routine governance functions through which it may be implemented (Maddox and Bradbrook 2025).

## Defining the object of intervention

### The commercial tobacco and nicotine industry as an implementable ecosystem

Implementation requires a precise definition of the object of intervention. In policy and regulatory contexts, abolition cannot proceed without an operational account of which commercial actors, products, infrastructures, and influence systems fall within the scope of reform. Rather than treating the industry as a singular actor, this framework understands the commercial tobacco and nicotine industry as an interconnected ecosystem comprising corporate structures, product categories, retail and supply chains, science and knowledge infrastructures, and political influence pathways (Maddox *et al*. 2026). Box 1 defines this broader ecosystem and distinguishes it from the narrower treaty definition of ‘tobacco industry’.

**Box 1** The commercial tobacco and nicotine industry as the object of intervention.

**Table:** 

The commercial tobacco and nicotine industry includes any entity engaged in the commercial manufacture, marketing, distribution, financing, or sale of tobacco or non-therapeutic nicotine products. This encompasses parent companies, subsidiaries, contractors, industry-funded research organizations, and third-party actors substantially controlled by, acting on behalf of, or collaborating with industry actors. Affiliates include entities that materially support, enable, finance, distribute, legitimize, protect, or extend commercial tobacco and nicotine industry operations, influence, supply chains, or market continuity. These entities may not directly manufacture or sell tobacco or nicotine products themselves, but nonetheless contribute to sustaining commercial industry function, expansion, political influence, or regulatory adaptation. Examples may include lobbying firms, trade associations, logistics providers, public relations agencies, legal firms, or industry-funded advocacy and research organizations. This definition excludes two distinct categories: sacred, ceremonial, or traditional tobacco; and nicotine products regulated as evidence-based pharmacotherapies. Commercial products described as ‘traditional’ or ‘traditionally manufactured’ cigarettes are distinct from sacred or ceremonial tobacco and should not be treated as equivalent. Where funding is provided pursuant to legal settlement, statutory obligation, or court order without industry control over its use, recipient entities are not classified as industry affiliates.

This box defines the commercial tobacco and nicotine industry as the object of intervention and establishes the regulatory boundary required for component-specific implementation, accountability, and enforcement.

This definition establishes the regulatory boundary required for component-specific implementation, accountability, and enforcement.

## Abolition by design: component-specific implementation

Abolition becomes implementable when the commercial tobacco and nicotine industry is disaggregated into component-specific sites of power, harm production, and regulatory intervention. Figure 1 provides the organizing architecture for the analysis that follows. It conceptualizes the industry as an interconnected implementation ecosystem comprising corporate structures and manufacturers; products and market systems; retail, distribution, and supply chains; science and knowledge infrastructures; political influence and policy interference; and growers, workers, and upstream supply chains.

**Figure 1:**
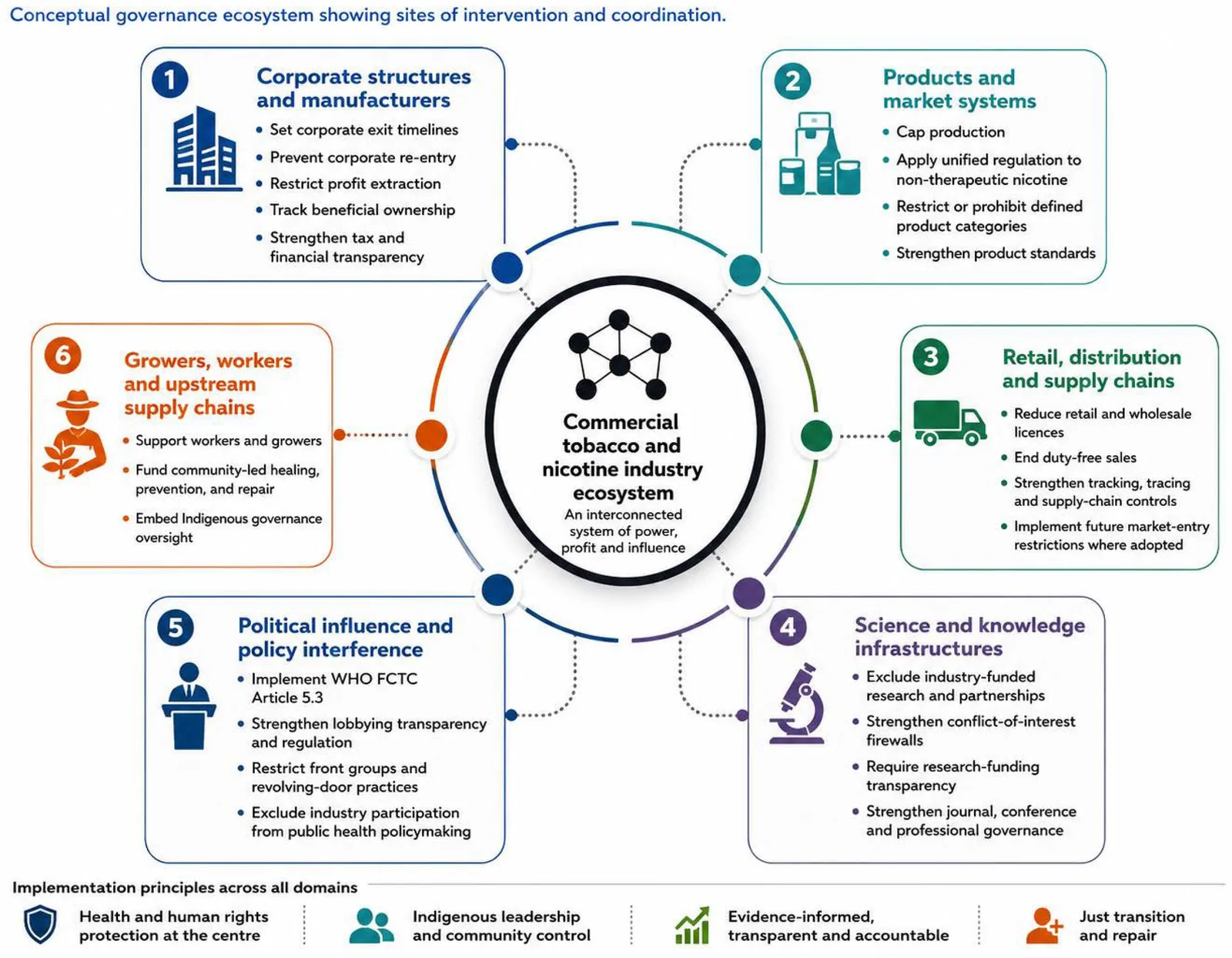
The commercial tobacco and nicotine industry as an interconnected implementation ecosystem. The ecosystem includes sites of intervention and coordination such as supply-chain monitoring, exclusion of industry partnerships from public institutions, and community-led reparative investment.

The subsections below explain how each domain sustains industry power and identify corresponding abolition and disruption levers. Table 1 then translates and extends this architecture into an operational mapping of system domains, actors, harms, abolition and disruption levers, legal and regulatory points of alignment, and lead institutions. A more detailed component-level mapping of abolition and disruption levers to relevant legal and regulatory provisions, international instruments, and responsible institutions is provided in Supplementary Table 1. Table 2 presents the phased sequencing of implementation across core industry functions. Although implementation is component-specific, action across several domains may occur in parallel to reduce regulatory gaps and limit opportunities for product substitution, corporate restructuring and regulatory arbitrage.

**Table 1:** The commercial tobacco and nicotine industry as an operational ecosystem: components, actors, and abolition levers.

	1. System domain	2. What this domain sustains	3. Core abolition and disruption levers	4. Primary legal anchors (WHO FCTC and related)	5. Lead institutions
1.	Corporate and ownership ecosystem	Concentrates power; shields liability; enables re-entry via subsidiaries, diversification, ESG ‘transition’ narratives	Corporate exit timelines; licence non-renewal; director disqualification; beneficial ownership transparency; profit extraction limits; mandatory divestment (where applicable)	Arts 5.3, 6, 19	Treasury; corporate regulators; courts; securities regulators; parliaments
2.	Product engineering and manufacturing	Designs addictiveness; sustains production scale; enables substitution across product categories	Production and profit caps; pre-market authorization; unified regulatory scope for non-therapeutic nicotine; category-based bans; manufacturing licence withdrawal; independent lab testing mandates	Arts 9–10, 15, 20	Health regulators; customs; standards bodies; enforcement agencies
3.	Access and supply infrastructure	Normalizes availability; embeds products in communities; sustains domestic and transnational distribution	Wholesale and retail licensing reduction; density and proximity caps; licence non-renewal; duty-free elimination; supply-chain due diligence; track-and-trace independence; equipment controls	Arts 6, 15, 16; Protocol Art 8	Local governments; customs; transport regulators; enforcement authorities
4.	Influence and narrative infrastructure	Manufactures doubt; launders legitimacy; enables policy interference through front groups and third parties	Conflict-of-interest firewalls; exclusion from policymaking; CSR/ESG exclusion policies; transparency registers; anti-SLAPP measures; permanent funding exclusion; platform-level TAPS enforcement	Arts 5.3, 12, 13, 19, 20	Integrity commissions; research councils; media regulators; justice ministries; professional bodies
5.	Financial and continuity ecosystems	Maintains profitability; arbitrages jurisdictions; enables reinvestment in harmful commercial activities; stabilizes investor confidence	Beneficial ownership disclosure; AML/CTF scrutiny; proceeds-of-crime actions; tax enforcement taskforces; banking and insurance due diligence standards; industry cost-recovery levies to fund regulatory oversight and enforcement	Arts 5.3, 15, 19	Tax authorities; financial intelligence units; securities regulators; central banks
6.	Structural, labour and colonial supply chain harms	Externalizes harm onto Indigenous peoples and racialized communities; entrenches economic dependency and environmental damage	Just-transition pathways; alternative livelihoods; modern slavery due diligence; reparative levies; Indigenous governance oversight; environmental remediation	Arts 17–18, 5.3, 19	Agriculture ministries; labour regulators; environment agencies; Indigenous governance bodies; development agencies

WHO FCTC provisions are presented as relevant points of alignment and are not direct or exhaustive legal authorizations for every measure listed. The availability and design of specific powers depend on applicable domestic, constitutional, and international law. Source: Framework informed by World Health Organization (2003), Gilmore *et al*. (2023), Maddox and Bradbrook (2025), and Maddox *et al*. (2026).

AML, anti-money laundering; CSR, corporate social responsibility; CTF, counter-terrorism financing; ESG, environmental, social, and governance; SLAPP, Strategic Lawsuit Against Public Participation; TAPS, tobacco advertising, promotion, and sponsorship; WHO FCTC, World Health Organization Framework Convention on Tobacco Control.

**Table 2:** Phased implementation of industry abolition across core industry functions.

Core industry function	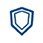 Phase 1: protect and insulate Shield policy, define scope, and establish foundations for action	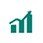 Phase 2: constrain and transition Constrain industry power and transition systems and communities	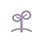 Phase 3: exit and repair End industry participation in markets and repair harms and systems
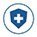 Policy insulation (Art 5.3, conflict-of-interest rules)	Adopt and enforce WHO FCTC Article 5.3 Establish conflict-of-interest rules and integrity safeguards	Strengthen lobbying and political influence restrictions Close loopholes; expand public transparency requirements	Exclude industry from policy forums permanently Embed safeguards in law and oversight institutions
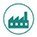 Profit and production constraints	Establish baselines for production, profits, and market share Introduce fiscal and price measures to reduce profitability	Impose production and profit caps Strengthen taxation and price escalation Restrict financial flows and close loopholes	Complete production phase-out Eliminate industry profit extraction from markets
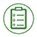 Licencing and compliance	Map licenses and authorizations Establish compliance and enforcement frameworks	Reduce licence numbers and tighten conditions Intensify compliance monitoring and enforcement	Do not renew licences Remove licences and authorizations from all industry actors
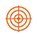 Density reduction	Establish baselines for retail and distribution density Halt expansion and new entrants	Reduce retail and wholesale density Restrict outlet location and proximity to sensitive settings	End duty-free and special access Remove remaining outlets and distribution channels
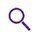 Scope definition	Define regulated products and actors Close regulatory gaps	Apply unified regulation across products and channels Phase-out non-therapeutic nicotine product categories	Complete category bans Update legislation to prevent (re)entry and circumvention
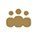 Transition planning	Engage affected communities and workers Co-design just-transition pathways	Implement alternative livelihoods and workforce support Provide social and economic support	Deliver long-term repair and community investment Advance intergenerational health and well-being

Implementation can follow a staged and coordinated approach. Phases are sequenced but non-exclusive; actions may proceed in parallel within and across phases, and timing will vary by jurisdiction.

Source: Framework informed by Maddox and Bradbrook (2025) and Maddox *et al*. (2026).

### Corporate structures and manufacturers

Corporate parent companies and manufacturing entities concentrate decision-making power, control production scale, and extract commercial returns from preventable harms (Legg *et al*. 2021, Gilmore *et al*. 2023). Abolition levers at this level include production and profit caps, non-renewal of manufacturing licences, mandatory transition plans, and clearly defined corporate exit timelines. Production caps refer to regulatory limits on the volume of tobacco and nicotine products permitted for manufacture or sale within a jurisdiction over time. Profit regulation may include excess-profit taxation, restrictions on shareholder distributions, or limits on commercial returns from harmful products. These measures are intended to reduce incentives for market expansion and continuing commercial dependence on nicotine addiction.

### Products and market systems

Product diversification has been a primary strategy through which the industry has adapted to regulation, shifting harms across combustible tobacco, e-cigarettes, synthetic nicotine, and other novel products. This diversification has enabled the continuation of commercial nicotine dependence through products frequently marketed as innovation, harm reduction, or public health transition. Abolition therefore requires unified regulatory scope definitions, category-based bans where appropriate, and the closure of synthetic nicotine loopholes. Treating non-therapeutic nicotine as a single regulatory class provides regulatory clarity and reduces opportunities for substitution and regulatory arbitrage.

### Retail, distribution, and supply chains

Retail and distribution networks determine commercial availability and deliver tobacco and nicotine products directly into communities. Licensing, density and proximity limits, compliance regimes, and not renewing licences can provide mechanisms through which availability may be progressively reduced (Pousini-Hilton *et al*. 2025). The Expert Group report identifies several forward-looking measures relevant to structural supply reduction, including retail reduction, the prohibition or phase-out of tobacco sales, ending the commercial sale of tobacco-industry products, and quotas on tobacco manufacture and imports followed by regular reduction (Expert Group on Forward-looking Tobacco Control Measures 2025). These are voluntary options whose design and implementation remain subject to the laws, contexts, and priorities of individual Parties.

A critical implication of this ecosystem perspective is that distinctions between legal and illegal tobacco and nicotine products, or between regulated and unregulated supply, should not obscure the broader commercial systems and demand environments that sustain nicotine availability and related harms (World Health Organization 2003, Legg *et al*. 2021, Crocetti *et al*. 2022, Gilmore *et al*. 2023). While some illicit tobacco and nicotine markets may operate independently of formal commercial actors, regulated and illicit markets frequently function through overlapping supply chains, product ecosystems, retail environments, and demand structures that sustain commercial nicotine availability. Examples include product substitution, adaptive distribution systems, and market environments shaped by commercial nicotine demand. Policy debates that position illegally traded products as a discrete crisis may risk narrowing reform towards enforcement cycles while leaving upstream commercial drivers intact. Within an abolition framework, the relevant policy objective is the progressive reduction of total commercial availability, regardless of regulatory status (Drescher 2009, Proctor 2012, Maddox and Bradbrook 2025). Addressing illicit supply is essential, but only as part of coordinated strategies that constrain commercial production, distribution, and industry influence across the commercial system as a whole (World Health Organization 2003, Colonna *et al*. 2020, Odo *et al*. 2025). Residual illicit tobacco and nicotine markets may still persist under abolition-oriented approaches, including through informal production or organized criminal networks, and would continue to require proportionate regulatory, enforcement, and public health responses.

Box 2 illustrates how regulated and unregulated products may circulate through overlapping commercial supply chains, retail environments, and demand systems, while recognizing that some illicit markets may also operate independently of formal commercial actors.

**Box 2** Regulated and unregulated trade within an interconnected commercial supply system.

**Table:** 

The distinction between regulated and unregulated trade reflects regulatory status but does not mean that these markets are wholly separate from the broader commercial systems that sustain tobacco and nicotine availability. Regulated and illicit markets may operate through overlapping supply chains, retail environments, product substitution and adaptive distribution pathways, although some illicit markets may also operate independently of formal commercial actors. As a result, illicit trade should not be understood solely as an external anomaly or the primary policy driver. Responses that focus narrowly on enforcement risk displacing harm while preserving upstream commercial drivers. Abolition reframes this challenge by prioritizing reduction of total commercial availability and addressing illicit supply as one expression of system resilience within a broader commercial model.

This box illustrates how regulated and unregulated tobacco and nicotine products may circulate through overlapping commercial supply chains, retail environments, and demand systems, while recognizing that some illicit markets may also operate independently of formal commercial actors.

### Science and knowledge infrastructures

Industry-funded research, consultancies, and professional influence pathways have played a central role in manufacturing doubt and delaying regulation (Oreskes and Conway 2012, Edwards *et al*. 2022). Abolition levers include permanent exclusion of industry actors and affiliates from public research funding, strengthened conflict-of-interest firewalls, and governance standards for journals, conferences, and professional bodies. These levers support implementation of WHO FCTC Article 5.3 and its implementation guidelines in relation to tobacco-industry interference, while protecting the integrity of evidence and knowledge systems (World Health Organization 2003, WHO Framework Convention on Tobacco Control 2013a). Comparable protections concerning nicotine-only commercial entities may be established through applicable domestic public health, integrity, and conflict-of-interest laws.

### Political influence and policy interference

Front groups, third-party allies, and revolving-door practices enable policy interference while obscuring industry accountability (Oreskes and Conway 2012, Legg *et al*. 2021). Transparency registers, exclusion from policymaking processes, lobbying restrictions, and cooling-off periods are established integrity mechanisms. Scrutiny of intellectual-property strategies that obstruct regulation, including trademark litigation, packaging-related legal challenges and product-design protections, may also reduce opportunities for regulatory delay and industry adaptation. When paired with monitoring, enforcement powers, and meaningful sanctions, these levers can help insulate public policy from commercial and vested interests.

### Growers, workers, and upstream supply chains

Abolition requires non-punitive exit pathways for workers and growers whose livelihoods have been shaped by commercial tobacco production. Just-transition measures, alternative-livelihood programmes, and land and labour protections align with WHO FCTC Articles 17 and 18 and the associated policy options and recommendations on economically sustainable alternatives to tobacco growing (World Health Organization 2003, WHO Framework Convention on Tobacco Control 2013b). These measures can shift power away from industry while protecting workers, growers, and communities from further harm.

Table 1 translates the conceptual ecosystem presented in Fig. 1 into an operational mapping of industry domains, actors, harms, abolition and disruption levers, legal and regulatory anchors, and lead institutions.

## Sequencing abolition: phased, parallel, and governable dismantling

Abolition is frequently misconstrued as requiring abrupt prohibition or immediate market closure (Department of Parliamentary Services 2026). In practice, effective abolition depends on sequencing: the deliberate ordering of policy actions to protect, insulate and stabilize governance, reduce industry power, and enable durable structural change over time (Drescher 2009, Proctor 2012, Maddox and Bradbrook 2025). Sequencing refers to phased progression over time and does not require that industry components be addressed one after another; multiple components may be acted upon in parallel within and across phases. Sequencing reduces political, legal, and economic risk while maintaining momentum towards industry exit (Maddox and Bradbrook 2025). In this sense, the implementation framework functions less as a static policy blueprint and more as a map for moving through time, guiding coordinated action across phases as systems shift, resist, and reconfigure (see Table 2; Maddox and Bradbrook 2025, Simpson 2025).

This phased logic illustrates how abolition-oriented reforms do not depend on any single policy intervention. Instead, durability emerges from coordinated tightening across domains, where early insulation measures (Article 5.3 implementation, conflict-of-interest firewalls) stabilize governance, enabling subsequent production constraints and eventual market exit (World Health Organization 2003, Maddox and Bradbrook 2025). Supply-side structural measures, such as Nicotine-Free Generation policies that progressively restrict commercial sales to specified birth cohorts, operate within Phase 2 as complementary mechanisms that progressively reduce market entry, reinforce long-term supply contraction, and shape social norms and demand trajectories (Ait Ouakrim *et al*. 2024, Maddox and Bradbrook 2025).

Table 2 demonstrates how coordinated interventions can progress across several industry domains without relying on a single policy act. Abolition, understood in this way, involves not only the withdrawal of harmful commercial structures but also the repair of relationships between people, land, knowledge systems, and governance that extractive systems have damaged over time (Maddox and Bradbrook 2025, Simpson 2025). Extended implementation and transition considerations, including just transition, reparative investment, environmental and labour protections, youth and community leadership, and adaptive governance, are provided in Supplementary Appendix B.

## Governance and institutional responsibility: whole-of-government action and Indigenous decision-making

Implementing abolition requires coordinated action across government portfolios and levels rather than reliance on health ministries alone. Regulatory, fiscal, enforcement, trade, and integrity institutions each hold responsibilities relevant to constraining industry power. The framework therefore combines whole-of-government coordination with Indigenous governance and decision-making, which is addressed as a distinct and cross-cutting requirement below.

### Whole-of-government leadership

Executive coordination across health, treasury, justice, trade, and regulatory portfolios is necessary for coherent implementation and to prevent policy displacement or exploitation of institutional silos (World Health Organization 2003, Heenan *et al*. 2024). Such coordination reflects WHO FCTC obligations and implementation commitments requiring multisectoral action and protection of public health policy from tobacco-industry interference, particularly under Articles 5.1 (multisectoral strategies), 5.2(a) (national coordination), and 5.3 (protection from industry interference) (World Health Organization 2003).

### Regulatory and enforcement authorities

Licensing authorities, customs, competition regulators, and consumer protection agencies provide the practical mechanisms through which abolition is enacted, monitored, and enforced. Strengthening these routine functions is central to addressing illicit tobacco and nicotine product trade, non-compliance, and industry interference (World Health Organization 2003, Heenan *et al*. 2024).

### Indigenous governance and decision-making

Indigenous governance structures should be embedded as decision-making authorities and oversight partners, rather than Indigenous peoples being treated as stakeholders consulted after policy priorities have been established. Indigenous peoples should hold authority to shape priorities, implementation pathways, accountability arrangements, and reparative investment, particularly in settler-colonial contexts where sovereignty, self-determination, and Indigenous rights are central governance considerations. This approach recognizes Indigenous peoples as rights holders and future makers with authority over healthy futures, rather than as populations requiring paternalistic protection. In other jurisdictions, abolition-oriented governance should similarly provide meaningful decision-making power to populations disproportionately affected by commercial tobacco and nicotine harms, while recognizing that Indigenous sovereignty and rights are not interchangeable with general stakeholder participation.

### International cooperation

Situating abolition within the WHO FCTC supports international policy coherence and collective reinforcement of ambitious structural reform through COP mechanisms under the WHO FCTC (World Health Organization 2003, Heenan *et al*. 2024).

## Feasibility, risk, and legal defensibility: implementing abolition using existing powers

Abolition is often characterized as politically or legally infeasible on the assumption that it requires extraordinary powers, unprecedented interventions, or a prohibitionist approach comparable to historical alcohol prohibition. Such comparisons often conflate abolition with consumer-level bans or prohibition, and overlook its focus on dismantling corporate production, supply, and profit structures through existing governance and regulatory tools (Proctor 2012, Fagerstrom *et al*. 2025, Maddox and Bradbrook 2025). This section addresses those concerns directly. By grounding abolition in existing regulatory, fiscal, and enforcement mechanisms, and aligning it with established obligations under the WHO FCTC and international human rights law, the framework illustrates how abolition-oriented reform may be administratively feasible and legally defensible within existing public health and governance systems (World Health Organization 2003, Proctor 2012, Ait Ouakrim *et al*. 2024, Maddox and Bradbrook 2025).

Box 3 summarizes the principal areas of alignment between abolition-oriented reform, the WHO FCTC and relevant international human rights instruments. It identifies legal and normative foundations for structural reform while recognizing that the availability and design of specific powers vary across jurisdictions. Detailed mapping of proposed abolition and disruption levers to relevant governance mechanisms, WHO FCTC provisions, Protocol to Eliminate Illicit Trade provisions, and responsible institutions is provided in Supplementary Appendix A.

**Box 3 Abolition and international legal alignment.** 

Abolition-oriented reform may be supported by, and aligned with, existing international public health and human rights standards. The WHO FCTC recognizes the fundamental conflict between the commercial interests of the tobacco industry and public health, and commits Parties to protect policy from industry interference, reduce demand and supply, address illicit trade, and consider legislative or other legal measures to address liability, including compensation where appropriate. Taken together, these provisions support progressively constraining industry power where it undermines population health. International human rights instruments, including the United Nations Declaration on the Rights of Indigenous Peoples (UNDRIP), affirm Indigenous peoples’ rights to self-determination, participation in decision-making in matters affecting their rights, the highest attainable standard of physical and mental health, and to determine and develop priorities and strategies for exercising their right to development. In contexts where a commercial industry continues to produce predictable and inequitable harms despite decades of regulation, abolition-oriented reform may be understood as consistent with, and potentially advancing, these commitments where persistent and preventable harms remain.

This box summarizes the principal areas of alignment between abolition-oriented reform, the WHO Framework Convention on Tobacco Control and relevant international human rights instruments. It identifies legal and normative foundations for structural reform while recognizing that the availability and design of specific powers vary across jurisdictions.

Source: World Health Organization (2003), United Nations (2007), Proctor (2012), Legg et al. (2021), Gilmore et al. (2023), Maddox and Bradbrook (2025), and Maddox et al. (2026).

### Political and institutional feasibility

Sequencing enhances feasibility by prioritizing early actions that protect policy from industry interference and reduce market power. Many proposed measures, including retail licensing and conflict-of-interest protections, build on regulatory approaches already familiar in public health and administrative governance. Other measures, including profit or production regulation, may require new or amended legislative authority depending on the jurisdiction (World Health Organization 2003, Ait Ouakrim *et al*. 2024, Maddox and Bradbrook 2025, Simpson 2025, Department of Parliamentary Services 2026).

### Legal and trade considerations

International experience indicates that strong tobacco-control measures can withstand legal and trade challenges when they pursue legitimate public health objectives, are supported by evidence, and are designed consistently with applicable domestic and international law. Alignment with WHO FCTC obligations can strengthen policy coherence, but legal defensibility must be assessed measure by measure and jurisdiction by jurisdiction. The framework therefore maps potential levers to existing legal foundations rather than assuming uniform authority across jurisdictions (World Health Organization 2003, Heenan *et al*. 2024).

### Industry adaptation and mitigation

The commercial tobacco and nicotine industry has a well-documented history of adapting to regulation through product substitution, corporate restructuring, and narrative reframing (Proctor 2012, Legg *et al*. 2021, Edwards *et al*. 2022, Maddox *et al*. 2026). Understanding the industry as an ecosystem and addressing multiple components in parallel may limit opportunities for regulatory arbitrage and reconstitution under new forms.

### Capacity and enforcement

While many abolition levers can operate through strengthening existing institutions and governance systems, some jurisdictions may require new institutional arrangements, coordination mechanisms, or expanded regulatory mandates depending on governance and constitutional context. Regardless of institutional configuration, abolition requires adequate resourcing, regulatory independence, and protection from industry interference. Over time, abolition may reduce long-term demand on health systems by preventing harms rather than attempting to manage or mitigate their consequences (Proctor 2012, Legg *et al*. 2021, Maddox and Bradbrook 2025).

## From implementation to durability: positioning abolition for long-term structural change

Durability depends on institutionalizing political, social, and cultural conditions that reduce opportunities for policy reversal, industry re-entry, and regulatory capture (Drescher 2009, Proctor 2012, Legg *et al*. 2021, Maddox and Bradbrook 2025). Public media campaigns, denormalization strategies, and abolition-oriented public communication may help strengthen public support and reduce the social legitimacy of commercial tobacco and nicotine industries (Malone and Proctor 2022). Embedding Article 5.3 protections, lobbying restrictions, transparency requirements, and conflict-of-interest safeguards within routine governance systems may further reduce the risks of policy capture and industry reconstitution.

Long-term stability may also be strengthened by embedding abolition objectives within broader public health, human rights, and intergenerational equity frameworks, making reversal politically and institutionally more difficult. The repeal of key Smokefree Aotearoa measures demonstrates the political fragility of ambitious tobacco endgame reforms and underscores the importance of sustained public support, Indigenous governance and accountability structures, and long-term institutional reinforcement (Andrew 2024).

## Conclusion

This paper translates abolition of the commercial tobacco and nicotine industry from a normative public health goal into a staged implementation framework. By defining the industry as an interconnected ecosystem, identifying component-specific sites of intervention and mapping these sites to existing governance, regulatory, and enforcement functions, the framework addresses the practical ‘how’ question that has often limited abolition-oriented debate.

Abolition, understood in this way, is not consumer prohibition or a single act of market closure. It is an upstream prevention strategy through which governments and communities can progressively constrain the structures that manufacture, distribute, and profit from tobacco- and nicotine-related harms, while protecting individuals, workers, and communities during transition. Indigenous governance, whole-of-government coordination, protection from industry interference, and institutional durability are central to this process.

Further work should develop abolition-aligned indicators, jurisdiction-specific legal pathways, enforcement ecosystems, and mechanisms that protect reforms against political reversal and industry reconstitution. By linking precise industry definition, component-specific interventions, and phased implementation, the framework positions abolition as a logical extension of longstanding tobacco control and health-promotion efforts to prevent harm at its commercial source.

## Supplementary Material

daag133_Supplementary_Data

## Data Availability

Not applicable.
