# Supplementary material for: Abolition by design: implementing the dismantling of the commercial tobacco and nicotine industry
Source: Health Promot Int. 2026 Oct 14;41(5):daag133. doi: 10.1093/heapro/daag133 (PMC13662213; doi:10.1093/heapro/daag133)
Supplement: Supplementary file 1 [file daag133_supplementary_data.zip › Supplementary_Appendix.doc]

Supplementary Appendix A. Legal and Regulatory Mapping

Supplementary Table 1 maps proposed abolition levers to relevant existing legal and regulatory provisions, international instruments and responsible institutions. These provisions represent potential points of alignment rather than direct or exhaustive legal authorisations for every measure identified. The availability and design of specific powers will vary by jurisdiction, and some measures may require new or amended legislative or regulatory authority.

Supplementary Appendix B. Extended Implementation Pathways and Transition Considerations
This appendix provides additional implementation considerations that support, but are not required to operationalise, the core abolition framework presented. It is intended as a practical resource for policymakers, implementers, and communities seeking to extend abolition beyond regulatory exit toward durable transition, repair, and prevention. These elements are therefore deliberately positioned as supplementary.
B1. Just Transition Pathways for Growers, Workers, and Retail-Dependent Communities
Abolition requires proactive planning to prevent the transfer of economic harms onto workers, small retailers, growers, and communities historically incorporated into tobacco supply chains. Just transition pathways may include:
Planned exit timelines aligned with licence non-renewal and production caps, providing predictability for affected actors.
Alternative livelihood programs for growers and upstream workers, building on existing FCTC Article 17 and 18 commitments and development-sector experience.
Workforce (re)deployment and (re)training, particularly for retail workers and logistics staff, prioritising secure employment in health-promoting or community-serving sectors.
Place-based transition strategies for regions with high retail density or historical tobacco and nicotine dependence.
B2. Reparative Investment and Community Repair Mechanisms

Beyond cessation of harms, abolition creates pathways for repair. Governments may consider:

Reparative levies or cost-recovery mechanisms applied to industry actors during transition phases, consistent with polluter-pays principles.
Targeted (re)investment in communities disproportionately harmed by commercial tobacco and nicotine, including Indigenous peoples and low-income communities.
Community-controlled allocation mechanisms to ensure investments reflect local priorities and sovereignty.
B3. Environmental and Labour Protections Across Supply Chains

Commercial tobacco and nicotine production has well-documented environmental and labour impacts. Implementation pathways may include:
Environmental remediation requirements tied to production and manufacturing exit.

Modern slavery and labour due-diligence obligations across remaining supply chains during transition.
Land restoration and protection measures, particularly where tobacco growing has contributed to soil degradation or water contamination.
B4. Youth and Community Leadership in Transition and Prevention

Abolition is strengthened when communities most affected by tobacco and nicotine harms are positioned as leaders rather than beneficiaries. Extended implementation considerations include:
Youth-led prevention and norm-setting initiatives, recognising young people as architects of future tobacco- and nicotine-free systems.
Formal roles for Indigenous governance structures in oversight, monitoring, and accountability during implementation and repair phases.

Community-based monitoring and feedback mechanisms to identify emerging risks, unintended consequences, or industry adaptation.
B5. Monitoring, Learning, and Adaptive Governance

Implementation pathways require governance systems capable of learning and adjustment over time. Key considerations include:
Independent monitoring bodies to track implementation fidelity, equity impacts, and industry responses.
Adaptive policy review cycles that allow tightening or recalibration of levers where harms persist.
Transparent public reporting to sustain legitimacy and public trust.

Together, these implementation pathways reinforce abolition as a governance implementation project oriented toward prevention, justice, and intergenerational health equity, while preserving the clarity and feasibility of the core implementation framework set out in the main paper.
