# Supplementary material for: Abolition by design: implementing the dismantling of the commercial tobacco and nicotine industry
Source: Health Promot Int. 2026 Oct 14;41(5):daag133. doi: 10.1093/heapro/daag133 (PMC13662213; doi:10.1093/heapro/daag133)
Supplement: Supplementary file 1 [file daag133_supplementary_data.zip › Supplementary_Table_1.doc]

Supplementary Table 1: The Commercial Tobacco and Nicotine Industry as a Governable System: Components, Actors, and Abolition Levers
Supplementary Table 1 maps proposed abolition levers to relevant existing legal and regulatory provisions, international instruments and

responsible institutions. These provisions represent potential points of alignment rather than direct or exhaustive legal authorisations for every measure identified. The availability and design of specific powers will vary by jurisdiction, and some measures may require new or amended legislative or regulatory authority.

Industry Component (explicit &
comprehensive)	What this component does / why it matters	Primary Abolition / Disruption Levers	WHO FCTC
Articles: relevant provisions
/ points of alignment	Protocol to Eliminate
Illicit Trade: relevant
provisions / points of
alignment	COP Guidelines / Decisions: relevant provisions / points of alignment	Responsible Agencies

/ Actors	
1. Parent companies,	Corporate

structuring that	Corporate exit

timelines; licence	Art 5.3,

Art 19	Offences & liability of	Art 5.3 Guidelines; COP decisions and	Corporate regulators; Treasury; courts;	


holding

structures & conglomerates	concentrates power, shields
accountability, and enables re-entry via subsidiaries.
Includes mergers and acquisitions, diversification, and re-entry via
adjacent product categories (e.g., "next-gen" and wellness/medical adjacencies).	non-renewal; restrictions on reconstitution;
dissolution where permitted	legal persons

(as transposed)	guidance on Article 19 (liability)	Competition/antitrust regulators	


2. Boards,

Chairpersons & Directors (incl. shadow directors)	Authorise strategy, interference,
diversification, and reputational
laundering.
Operates through board committee
infrastructure (audit and risk,
remuneration, ESG/sustainability, and sometimes
science/medical advisory structures) that
institutionalises	Director

disqualification; fit-and-proper tests;
exclusion from

policymaking; civil liability pathways;
Committee-level transparency and accountability
(audit/risk/ESG) including disclosure of advisers and third-party relationships	Art 5.3,

Art 19	Art 15 legal-person
liability;
individual

director/officer liability
subject to

national law	Art 5.3 Guidelines

(conflicts/interaction limits); COP
decisions and

guidance on Article 19	Corporate regulators; securities
commissions; courts	


reputational and

regulatory strategy.	
3. Shareholders	Provide capital;	Beneficial ownership	Art 5.3,	Indirect /	Art 5.3 Guidelines;	Financial regulators;	
& beneficial	exert pressure to	disclosure;	Art 19	jurisdiction-	COP decisions and	stock exchanges;	
owners	maintain profit	dividend/profit		dependent	guidance on Article	Treasury	
	extraction; enable	extraction limits;		(including	19 (liability)		
	ESG “transition”	compulsory		through			
	narratives	divestment pathways		proceeds-of-			
				crime and			
				legal-person			
				liability			
				provisions)			
4. Senior	Execute strategy;	Executive	Art 5.3,	—	Art 5.3 Guidelines;	Integrity bodies;	
executives &	coordinate	accountability;	Art 19		COP decisions and	corporate regulators	
management	lobbying, market	cooling-off periods;			guidance		
	expansion, and	exclusion from					


	regulatory evasion. Uses succession planning, executive churn, and role
reconfiguration to maintain continuity during regulatory
shocks.	advisory roles;

Executive

remuneration

constraints linked to volume,
addictiveness, and youth uptake metrics					
5.

Manufacturers (combustible & non-combustible)	Produce addictive products; control production scale and product design	Production & profit caps; manufacturing licence withdrawal; product bans	Arts 9–10,

15, 16, 19	Arts 6–9

(licensing, due diligence,
T&T, records)	Partial Arts 9–10

Guidelines; Protocol implementation
guidance	Health regulators;

customs; enforcement agencies	
6. Brand & product	Enables regulatory arbitrage and	Unified scope

definition; category-based bans; closure of	Arts 9–10,

11, 13	—	Arts 9–10, 11 & 13

Guidelines; COP10 cross-border TAPS	Health ministries; standards bodies	


portfolios (incl. “next-gen”)	substitution across product categories	synthetic nicotine loopholes					
7. Marketing, advertising, promotion & sponsorship (incl. digital)	Drives uptake;

normalises harm; circumvents
national borders	Comprehensive TAPS bans; influencer &
digital platform
regulation; AdTech and data-broker
restrictions; affiliate marketing bans; platform duties and ad repository/audit
requirements; cross-border digital enforcement MOUs	Art 13, Art

12	—	Art 13 Guidelines; COP decisions and guidance on cross-border and digital TAPS	Media regulators; health ministries; Privacy regulators; platform
regulators/eSafety-style agencies	
8. Packaging, labelling &	Pack functions as advertising and	Plain packaging;

design restrictions;	Art 11, Art

13	—	Art 11 & 13 Guidelines	Health ministries; consumer regulators	


“design as marketing”	misleads

consumers; pack signals licit and illicit trade of products	disclosure

requirements					
9. Retailers	Normalise	Retail licensing	Art 16, Art	Art 11	Art 15 FCTC text;	Local governments;	
(physical &	availability;	reduction; licence	15		Protocol guidance	enforcement agencies	
online)	facilitate youth	non-renewal; tiered					
	access, informal	retail licensing with					
	supply, and price	escalating fees;					
	discounting; act as	density and proximity					
	the final point of	caps; strict					
	commercial harm	compliance regimes;					
	delivery into	administrative fines					
	communities	proportionate to					
		turnover; licence					

suspension and non-renewal; civil
penalties for repeat breaches; criminal penalties for large-scale or organised non-compliance; mandatory exit
timelines for tobacco retail; prohibition on licence transfer or
resale; cost-recovery levies to fund enforcement and
community repair


10.

Wholesalers, distributors & logistics providers	Maintain

distribution

resilience; enable parallel supply
routes	Licensing; due

diligence; freight & parcel controls	Art 15	Arts 6–9

(licensing,

T&T, records)	Protocol text & COP implementation
decisions	Customs; transport regulators	
11. Importers, exporters, brokers &
freight
forwarders	Facilitate

transnational flows; mis-declaration	Licensing; cooperation
agreements; penalties	Art 15	International transit, free zones,
cooperation	Protocol obligations	Customs; trade authorities	
12. Free zones & international transit
operators	Create “grey

channels” outside effective oversight	Prohibition or strict

regulation of tobacco in free zones	Art 15	Art 12 (free zones &
transit)	Protocol guidance	Customs; trade ministries	


13. Duty-free sales channels	Undermine

price/tax measures; leakage into
domestic markets	Elimination of duty-free tobacco; tax
harmonisation	Art 6, Art

15	Art 13 (duty-free sales)	Art 6 Guidelines; COP tax decisions	Treasury; customs	
14.

Manufacturing equipment suppliers	Enable illicit and unlicensed production	Licensing; equipment tracking; export controls	Art 15	Supply-chain controls (as defined
nationally)	Protocol text	Customs; industry regulators	
15. Growers & upstream supply chains	Entrench

dependency; labour and environmental harms	Just transition; alternative
livelihoods; land protections	Arts 17–

18	—	COP work on alternatives to
tobacco growing	Agriculture;

development agencies	
16. Science distortion
infrastructure
(funders,	Launder evidence; manufacture doubt. Includes standards-
setting, contract	Funding exclusion; COI firewalls;
permanent exclusion;
Mandatory register of	Art 5.3,

Art 20	—	Art 5.3 Guidelines	Research councils; universities; Standards	


institutes, consultancies)	research

organisations, testing labs,
regulatory science submissions,
guideline influence, and methodological capture.	industry-funded

research; independent methodological
audits; standards-setting COI
exclusions				bodies; independent laboratories	
17. Professional influence pathways (journals,
conferences, training)	Normalise industry participation in
health spaces	Sponsorship bans; disclosure &
exclusion rules	Art 5.3	—	Art 5.3 Guidelines	Professional bodies; journals	


18. Front groups &
third-party allies (“astroturf”)	Proxy lobbying and policy interference	Transparency

registers; exclusion from consultations	Art 5.3	—	Art 5.3 Guidelines	Integrity commissions	
19. Lobbying, political donations & revolving doors	Capture

policymaking; delay regulation	Lobbying bans;

cooling-off periods; disclosure	Art 5.3	—	Art 5.3 Guidelines	Parliaments; ethics bodies	
20. Illicit manufacturing networks	Counterfeit and unlicensed production	Criminal penalties; equipment seizure	Art 15	Offences;

legal-person liability	Protocol text	Law enforcement	
21. Illicit

distribution networks	Informal markets; social supply	Cooperation; enforcement; demand reduction	Art 15	Cooperation & enforcement	Protocol text	Law enforcement; customs	


22. Money flows (tax evasion, laundering, proceeds)	Sustain profitability;
reinvest harm.
Includes transfer pricing, internal
reinsurance/captive finance, and
related-party
transactions used to extract profit and
shift risk across jurisdictions.	Financial

surveillance;

proceeds-of-crime actions; Beneficial ownership and
related-party transaction
transparency; tax enforcement taskforces	Art 15, Art

19	Cooperation & offences	COP decisions and guidance on Article 19; Protocol
cooperation	Financial intelligence units; Tax authorities; securities regulators	
23. Litigation, intimidation & strategic lawsuits	Chill regulation;

deter public interest action	Legal defence funds; anti-Strategic
Lawsuits Against	Art 5.3,

Art 19	—	COP decisions and guidance on Article 19 (liability)	Justice ministries; courts	


Public Participation (SLAPP) measures	
24. Corporate	Designs litigation	Mandatory disclosure	Art 5.3,	Offences and	Art 5.3 Guidelines;	Justice/Attorney-	
legal,	survival, drafts	of external	Art 19	liability of	COP decisions and	General; integrity	
compliance and	"responsible	counsel/consultants		legal persons	guidance on Article	bodies; corporate	
strategic	regulation"	on regulatory		(as	19 (liability) stream	regulators;	
regulation	templates, builds	submissions;		transposed);		procurement	
apparatus (in-	symbolic	debarment and		cooperation		authorities; courts	
house and	compliance, and	procurement		and records			
external)	shapes	exclusion for repeat		(where			
	administrative law	interference; duty-to-		applicable)			
	risk to delay,	cooperate with					
	weaken, or	regulators; anti-					
	fragment	obstruction penalties;					
	regulation.	cost orders and					
		contempt					


enforcement; anti-SLAPP measures; strengthened
corporate liability and director/officer
accountability pathways.	
25. PR,	Converts harm into	CSR/ESG exclusion	Art 5.3,	—	Art 5.3 Guidelines	Integrity commissions;	
reputation,	legitimacy, opens	policies across	Art 13		(avoid	procurement;	
CSR/ESG and	doors to health and	government,			partnerships/CSR);	universities; charities	
transition	equity spaces,	universities, and			Art 13 Guidelines	regulators; consumer	
laundering	recruits validators,	NGOs; transparency				regulators	
infrastructure	and stabilises	registers for CSR and					
	investor confidence	third-party payments;					
	through CSR/ESG	bans on sponsorship					
	narratives and	and partnerships;					


	"transformation" claims.	charity and university COI firewalls;
debarment from
public funding and tenders.					
26. Digital	Enables	Platform duties and	Art 13, Art	—	Art 13 Guidelines;	Media/communications	
marketing	microtargeting,	enforceable TAPS	12		COP decisions on	regulators; privacy	
supply chain	youth adjacency,	bans; ad repository			cross-border/digital	regulators; consumer	
(AdTech, data	cross-border	and audit rights; bans			TAPS	protection; platform	
brokers,	promotion, and	on paid amplification				regulators	
platforms,	ineffective age-	and influencer					
influencers,	verification	marketing; affiliate					
affiliates)	practices by	marketing bans; data-					
	outsourcing	broker restrictions					
	marketing to data	and youth profiling					
		prohibitions; cross-					


	and platform ecosystems.	border enforcement MOUs and
cooperation.					
27. Product	Engineers	Pre-market	Arts 9–10,	—	Partial Arts 9–10	Health regulators;	
design, R&D,	addictiveness and	authorisation with	Art 20, Art		Guidelines; Art 5.3	standards bodies; IP	
patents and	appeal, uses IP to	public-interest tests;	5.3		Guidelines	offices; independent	
standards	lock in market	product standards and				laboratories	
manipulation	power, and shapes	bans; mandatory					
(including	what regulators can	disclosure of					
nicotine	measure through	ingredients,					
engineering)	standards and	emissions, and design					
	testing methods.	features; independent					
		lab testing mandates;					
		COI exclusions from					
		standards-setting;					
		scrutiny of IP					


practices that obstruct regulation.	
28. Finance	Maintains	Enhanced AML/CTF	Art 15, Art	Cooperation	Protocol	Treasury; tax offices;	
(treasury,	profitability and	scrutiny; beneficial	5.3, Art 19	and offences	cooperation	financial intelligence	
transfer	continuity,	ownership and		(as	decisions; COP	units; securities	
pricing, tax	arbitrages	related-party		implemented);	decisions and	regulators	
structuring and	jurisdictions, funds	transaction		proceeds-of-	guidance on Article		
investor	interference, and	transparency;		crime linkages	19 (liability); Art 5.3		
relations)	manages debt,	restrictions on profit			Guidelines		
	dividends, and	extraction where					
	capital discipline.	permitted; tax					
		enforcement					
		taskforces; disclosure					
		of political and third-					
		party spend to					
		investors; sanctions-					


style restrictions for repeat corporate
offenders (where lawful).	
29. Banking,	Keeps operations	Sectoral de-risking	Art 15, Art	Cooperation;	Protocol	Financial regulators;	
insurance and	bankable and	standards; enhanced	5.3, Art 19	financial	implementation	insurance regulators;	
payment rails	insurable, enables	due diligence;		investigations	guidance; Art 5.3	FIUs; payments	
(commercial	payments for legal	restrictions on		and offences	Guidelines	regulators	
services	and illegal supply,	underwriting and		(as			
enabling	and supports	broking; payment		implemented)			
continuity)	resilience when	platform rules and					
	other nodes are	merchant monitoring;					
	disrupted.	anti-money					
		laundering					
		investigations and					


asset restraint where appropriate.	
30. State-linked ownership and state trading interests (where
applicable)	Creates policy

incoherence and conflicts when
government is an

owner, beneficiary, or privileged
trading partner,

weakening Article 5.3
implementation.	Mandated divestment pathways; conflict-of-interest firewalls;
governance separation;
transparency on state revenue flows and
decision pathways; exclusion of SOE
tobacco entities from policy participation.	Art 5.3,

Art 6	—	Art 5.3 Guidelines	Treasury; SOE regulators;
parliaments; integrity bodies	
31. Labour, racialised harms and	Externalises harm onto colonised and racialised peoples	Human rights and modern slavery due
diligence; enforceable	Arts 17–

18, Art
5.3, Art 19	—	COP work on alternatives to	Labour regulators; human rights
commissions;	


colonial supply-chain impacts	through land, labour exploitation, environmental
damage, and targeted market practices;
perpetuates
structural violence and dispossession.	supply-chain transparency;
reparative levies and community repair
mechanisms; procurement exclusions; environmental enforcement;
Indigenous data sovereignty
requirements for monitoring and accountability.			tobacco growing; Art 5.3 Guidelines	environment agencies; agriculture; Indigenous affairs; procurement authorities	
32. Tracking
and tracing	Provide systems
and infrastructure	Government-
controlled or	Art 15, Art
5.3	Protocol Art 8
(tracking and	Protocol
implementation	Customs; health
ministries;	


and compliance technology
vendors
(marks, codes, databases, audit tools)	for product movement
visibility; can be used to strengthen enforcement, but also to create
dependency or
enable interference if industry-linked
(e.g., control over data, standards, or access).	independent track-and-trace
procurement; prohibition on
industry ownership or control of T&T
systems; open
technical standards; mandatory
independent security and governance
audits; data access rights for enforcement;
procurement

debarment for	tracing);

cooperation and information sharing	guidance; Article

5.3 Guidelines

(avoiding industry-linked systems)	digital/government

services; procurement authorities; data
protection regulators; law enforcement
